# Supplementary material for: Integrated clinical and genomic analysis identifies driver events and molecular evolution of colitis-associated cancers
Source: Nat Commun. 2023 Jan 7;14:110. doi: 10.1038/s41467-022-35592-9 (PMC9825391; doi:10.1038/s41467-022-35592-9)
Supplement: Supplementary file 1 — Supplementary Information [file 41467_2022_35592_MOESM1_ESM.pdf]

Supplementary Table 1. Germline alterations detected in patients with CAC

Gene affected	Alteration	Number of patients	Variant Classification (ACMG)[1]	Variant Classification (IARC)[2]
APC	c.3920T>A (p.Ile1307Lys)	3	Pathogenic	5
PMS2	c.943C>T (p.Arg315*)	1	Pathogenic	5
PMS2	c.137G>T (p.Ser46Ile)	1	Pathogenic	5
FANCA	exon 9-23 deletion	1	Pathogenic	5
DICER1	c.4972delA (p.Thr1658Hisfs*2)	1	Pathogenic	5
ATM	c.7875_7876delinsGC (p.AspAla2625GluPro)	1	Likely pathogenic	4
FANCC	c.456+4A>T ()	1	Pathogenic	5
RAD51B	c.321delA (p.Gly108Valfs*12)	1	Likely pathogenic	4

Supplementary Table 2. Beta-catenin Immunohistochemistry in CAC		
Patient ID	Wnt pathway alteration	CTNNB1 staining
P-0039752	APC MUT	5% NUCLEAR
P-0045221	WT	MEMBRANOUS
P-0042542	WT	MEMBRANOUS
P-0045240	APC MUT	15% NUCLEAR
P-0022336	WT	MEMBRANOUS
P-0022822	WT	MEMBRANOUS
P-0020135	APC MUT	MEMBRANOUS
P-0025046	WT	MEMBRANOUS
P-0028913	WT	60% NUCLEAR
P-0035913	RNF43 MUT	MEMBRANOUS
P-0014499	WT	MEMBRANOUS
P-0014021	WT	MEMBRANOUS
TRF094958	WT	MEMBRANOUS
P-0011396	APC MUT	50% NUCLEAR

Abbreviations: WT: wild-type; MUT: mutated

Supplementary Table 3. M-IMPACT V2 Gene List

Abl1	Bard1	Cdkn2b	Egr1	Fbxo11	Hras	Klhl9	Mob3b	Nxf1	Prdm14	Rptor	Smarcd1	Tgfbr1
Actg1	Bbc3	Cdkn2c	Egr2	Fbxw7	Icosl	Kmt2a	Mpeg1	Pak1	Prex2	Rragc	Smarce1	Tgfbr2
Acvr1	Bcl2	Cebpa	Eif1ax	Fgf3	Id3	Kmt2b	Mpl	Palb2	Prkar1a	Rras	Smc1a	Tmem127
Ago1	Bcl2l1	Cenpa	Eif4a2	Fgf4	Idh1	Kmt2c	Msh2	Parp1	Prkcb	Rras2	Smc3	Tmprss2
Ago2	Bcl2l11	Chd2	Eif4e	Fgf15	Idh2	Kmt2d	Msh3	Pax5	Prkci	Rtel1	Smg1	Tnfaip3
Akt1	Bcl6	Chek1	Elf3	Fgfr1	Ifna1	Kmt5a	Msh6	Pbrm1	Prkd1	Runx1	Smo	Tnfrsf1b
Akt2	Bcl10	Chek2	Ep300	Fgfr2	Ifna4	Knstrn	Msi1	Pcbp1	Ptch1	Runx1t1	Smyd3	Tnfrsf14
Akt3	Bcl11b	Cic	Ep400	Fgfr3	Ifnb1	Kras	Msi2	Pdcd1	Pten	Rxra	Socs1	Top1
Alb	Bcor	Ciita	Epas1	Fgfr4	Ifne	Ksr2	Mst1	Pdcd1lg2	Ptp4a1	Rybp	Socs2	Traf2
Alk	Bcorl1	Cmtr2	Epcam	Fh1	Ifngr1	Lats1	Mst1r	Pdgfra	Ptpn1	Samhd1	Socs3	Traf3
Alox12b	Bcr	Crbn	Epha3	Flcn	Igf1	Lats2	Mtap	Pdgfrb	Ptpn2	Sbds	Sos1	Traf5
Amer1	Birc3	Crebbp	Epha5	Flt1	Igf1r	Lck	Mtor	Pdpk1	Ptpn11	Scg5	Sox2	Traf7
Ankrd11	Blm	Crkl	Epha7	Flt3	Igf2	Lmo1	Mutyh	Pds5a	Ptprd	Sdha	Sox9	Trip13
Apc	Bmpr1a	Crlf2	Ephb1	Flt4	Ikbke	Lrp1b	Mybbp1a	Pds5b	Ptprs	Sdhaf2	Sox17	Trp53
Aplnr	Braf	Csde1	Erbb2	Foxa1	Ikzf1	Ltb	Myc	Pgbd5	Ptprt	Sdhb	Sp140	Trp53bp1
Ar	Brca1	Csf1r	Erbb4	Foxf1	Ikzf3	Lyn	Mycn	Pgr	Rab35	Sdhc	Spen	Trp63
Araf	Brca2	Csf3r	Ercc2	Foxl2	Il7r	Lztr1	Myd88	Phf6	Rac1	Sdhd	Spop	Tsc1
Arhgap35	Brd4	Ctcf	Ercc3	Foxo1	Il10	Mad2l2	Myo18a	Phip	Rac2	Serp2	Spred1	Tsc2
Arhgef28	Brip1	Ctla4	Ercc4	Foxo3	Inha	Malt1	Myod1	Phox2b	Rad21	Serpinb3a	Sprtn	Tshr
Arid1a	Btg1	Ctnnb1	Ercc5	Foxp1	Inhba	Map2k1	Nadk	Piga	Rad50	Serpinb3b	Spry4	Tyk2
Arid1b	Btk	Ctr9	Erf	Fubp1	Inpp4a	Map2k2	Nbn	Pik3c2g	Rad51	Sesn1	Src	U2af1
Arid2	Calr	Cul3	Erg	Furin	Inpp4b	Map2k4	Ncoa3	Pik3c3	Rad51b	Sesn2	Srcap	U2af2
Arid3a	Card11	Cux1	Errfi1	Fyn	Inppl1	Map3k1	Ncor1	Pik3ca	Rad51c	Sesn3	Srp72	Ubr5
Arid3b	Carm1	Cxcr4	Esco1	Gab1	Insr	Map3k6	Ncor2	Pik3cb	Rad51d	Setbp1	Srsf2	Upf1
Arid3c	Casp8	Cyld	Esco2	Gab2	Irf1	Map3k7	Ncstn	Pik3cd	Rad52	Setd1b	Stag1	Usp8
Arid4a	Cbfb	Cyp19a1	Esr1	Gata1	Irf4	Map3k13	Negr1	Pik3cg	Rad54l	Setd2	Stag2	Vav1
Arid4b	Cbl	Daxx	Etaa1	Gata2	Irf8	Map3k14	Nf1	Pik3r1	Raf1	Setd3	Stat3	Vav2
Arid5a	Cblb	Dcun1d1	Etnk1	Gata3	Irs1	Mapk1	Nf2	Pik3r2	Rara	Setd4	Stat5a	Vegfa
Arid5b	Ccnd1	Ddr2	Etv1	Gli1	Irs2	Mapk3	Nfe2l2	Pik3r3	Rasa1	Setd5	Stat5b	Vhl
Asxl1	Ccnd2	Ddx3x	Etv4	Gna11	Itpkb	Mapkap1	Nfkbia	Pim1	Rb1	Setd6	Stat6	Vtcn1
Asxl2	Ccnd3	Ddx41	Etv5	Gna12	Jak1	Max	Nipbl	Plcg1	Rbm10	Setd7	Stk11	Wt1
Atm	Ccne1	Dicer1	Etv6	Gna13	Jak2	Mcl1	Nkx2-1	Plcg2	Recql	Setdb1	Stk19	Wwtr1
Atp6ap1	Cd28	Dis3	Exosc6	Gnas	Jak3	Mdc1	Nkx3-1	Plk1	Recql4	Setdb2	Stk40	Xbp1
Atp6v1b2	Cd70	Dnajb1	Ezh1	Gnb1	Jarid2	Mdm2	Notch1	Plk2	Rel	Sf3b1	Sufu	Xiap
Atr	Cd79a	Dnmt1	Ezh2	Gps2	Jun	Mdm4	Notch2	Pmaip1	Rest	Sgk1	Suz12	Xpo1
Atrx	Cd79b	Dnmt3a	Faf1	Grem1	Junb	Mecom	Notch3	Pms1	Ret	Sh2b3	Syk	Xrcc2
Atxn2	Cd274	Dnmt3b	Fanca	Grin2a	Kbtbd4	Med12	Npm1	Pms2	Rheb	Sh2d1a	Tap1	Yap1
Atxn7	Cd276	Dot1l	Fancc	Gsk3b	Kdm5a	Mef2b	Nras	Pnrc1	Rhoa	Shoc2	Tap2	Yes1
Aurka	Cdc42	Drosha	Fancd2	Gtf2i	Kdm5c	Men1	Nsd1	Pold1	Rhoh	Shq1	Tbl1xr1	Ylpm1
Aurkb	Cdc73	Dtx1	Fance	Hdac1	Kdm6a	Met	Nt5c2	Pole	Rictor	Slx4	Tbx3	Zeb2
Axin1	Cdh1	Dusp1	Fancf	Hdac4	Kdr	Mga	Nthl1	Pot1a	Rit1	Smad2	Tcf3	Zfhx3
Axin2	Cdk4	Dusp4	Fancg	Hdac7	Keap1	Mgam	Ntrk1	Pot1b	Rnf43	Smad3	Tcf7l2	Zfhx4
Axl	Cdk6	Dusp22	Fanci	Hdac8	Kit	Mib1	Ntrk2	Pparg	Robo1	Smad4	Tek	Zfp292
B2m	Cdk8	E2f3	Fancl	Hgf	Klf2	Mitf	Ntrk3	Ppm1d	Ros1	Smarca1	Tert	Zfp318
Babam1	Cdk12	Eed	Fancm	Hif1a	Klf4	Mki67	Nuf2	Ppp2r1a	Rps6kb2	Smarca2	Tet1	Zmym3
Bach2	Cdkn1a	Egfl7	Fas	Hnf1a	Klf5	Mlh1	Nup93	Ppp6c	Rps15	Smarca4	Tet2	Znrf3
Bap1	Cdkn1b	Egfr	Fat1	Hoxb13	Klhl6	Mllt1	Nup98	Prdm1	Rps19	Smarcb1	Tet3	Zrsr1

Supplementary Table 3: Mouse-IMPACT version 2 gene list

Supplementary Figure 1

a

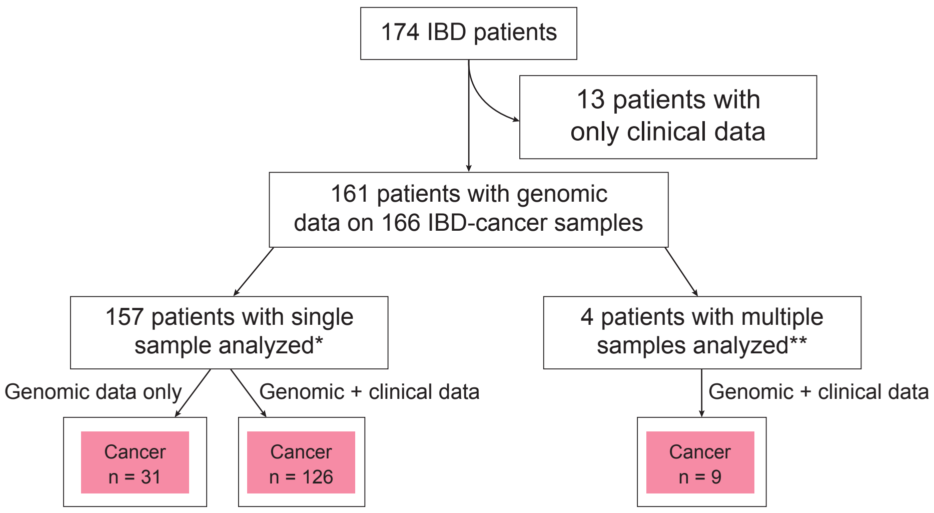

*includes 45 CAC patients from Yaeger et al, Gastroenterology 2016[3]
**includes 2 CAC patients from Yaeger et al, Gastroenterology 2016[3]

b

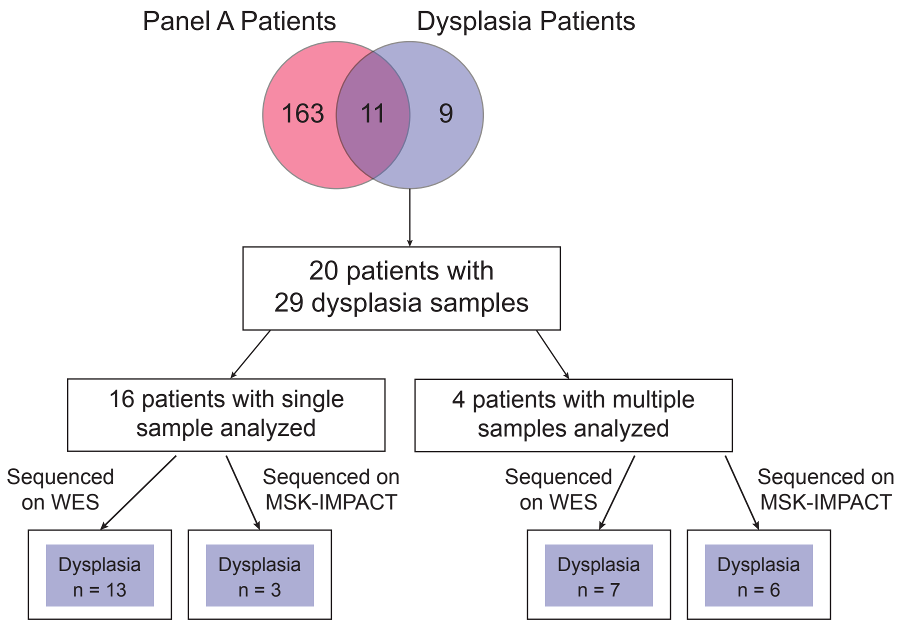

Supplementary Fig. 1. Cohort overview and sample breakdown.
a, Flow diagram showing number of CAC patients analyzed for clinical and molecular characteristics. **b,** Flow diagram showing IBD patients whose dysplasia samples were analyzed and sequencing assay used.
Abbreviations: WES: whole-exome sequencing.

Supplementary Figure 2

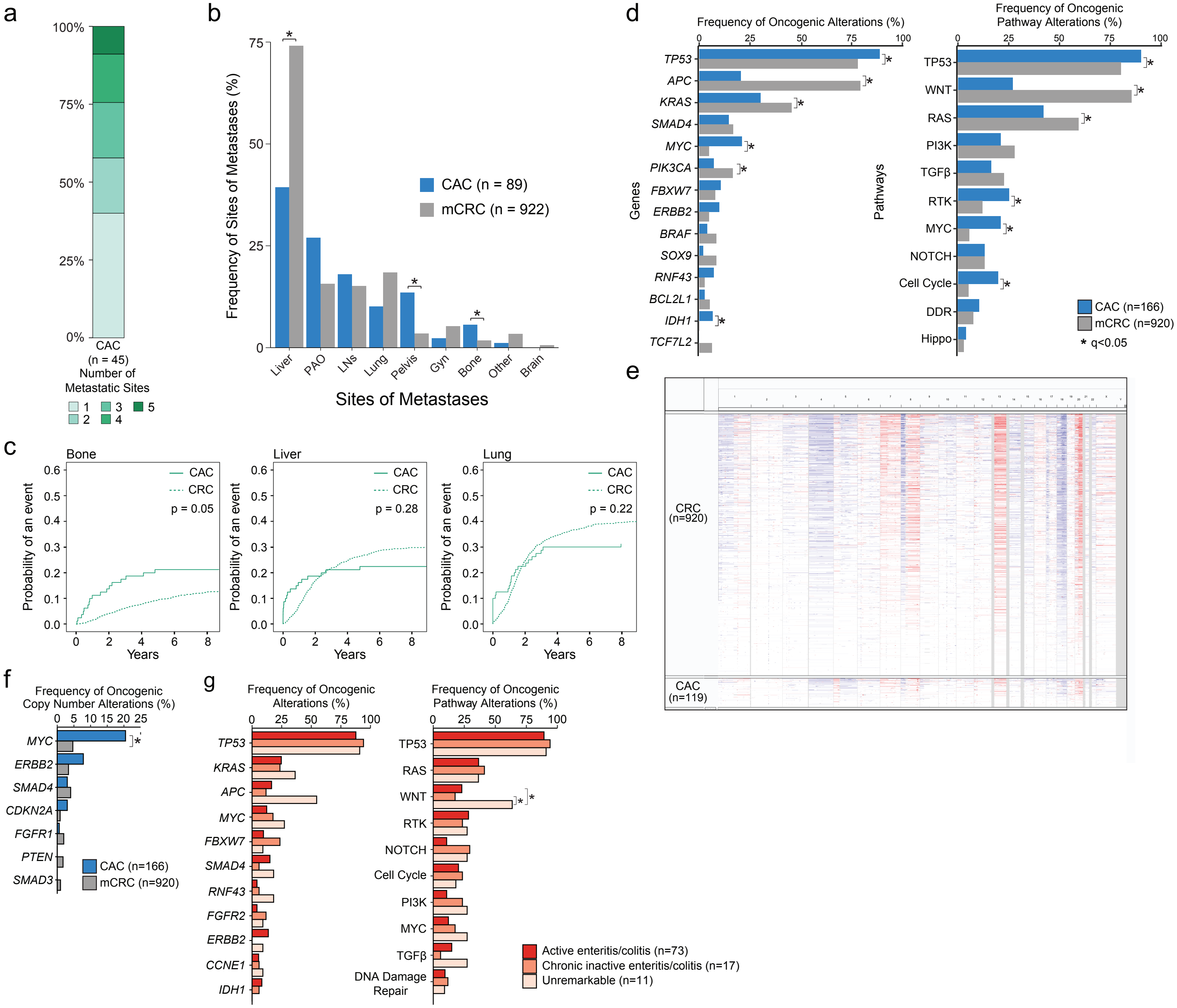

Supplementary Fig. 2. Comparison of metastatic patterns and genomics in CAC versus CRC.
a, Number of sites of metastases in patients with CAC at time of metastatic diagnosis. **b,** Metastatic sites at time of metastasis diagnosis in CAC (n = 89) and CRC (n = 922) patients. Pelvis (P = 0.019) and bone (P = 0.031) metastases were enriched in CAC, while liver metastases (P = 5.3e-14) were less common in CAC. **c,** Cumulative incidence curves for bone, liver, and lung metastases in patients where these sites were not involved at time of metastasis diagnosis. **d,** Recurrent oncogenic gene and pathway alterations. In CAC cases compared to CRC, alterations in *TP53* (89.8% versus 78.0%, P = 0.00032, q = 0.011), *MYC* (20.5 versus 4.8%, P = 3.5e-10, q = 3.5e-8), and *IDH1* (6.6% versus 0.1%, p = 8.3e-9, q = 5.5e-7) were significantly enriched, whereas *APC* (20.5% versus 77.7%, P = 2.4e-45, q = 4.8e-43), *KRAS* (30.7% versus 45.5%, P = 4.6e-8, q = 1.3e-2), and *PIK3CA* (6.6% versus 16.5%, P = 5.6e-4, q = 1.4e-2) were significantly less frequent. Additionally, P53 (91.0% versus 80.3%, P = 6.4e-4, q = 1.4e-3), RTK (25.9% versus 12.1%, P = 1.1e-5, q = 3.4e-5), MYC (20.5% versus 5.7%, P = 8.4e-9, q = 5.4e-8), and cell cycle (19.3% versus 5.2%, P = 1.8e-8, q = 7.8e-8) pathways were enriched in CAC, whereas WNT (27.7% versus 83.8%, P = 2.9e-46, q = 3.8e-45) and RAS (41.6% versus 59.5%, P = 2.7e-5, q = 7.1e-5) pathways were enriched in CRC. **e,** Copy number profiles. **f,** Oncogenic focal copy number alterations. *MYC* alterations were higher in CAC versus CRC (P = 2.26e-10, q = 1.58e-9). **g,** Genomic alterations (left) and pathways altered (right) in CAC grouped by appearance of non-neoplastic mucosa within the resected bowel. WNT pathway was significantly enriched in the unremarkable group compared to the active enteritis/colitis (63.6% versus 23.0%, P = 0.0098, q = 0.13) and chronic inactive enteritis/colitis (63.6% versus 17.6%, P = 0.020, q = 0.26) groups. Comparisons performed using two-sided Fisher's exact test. Statistically significant results indicated with asterisk. Source data are provided as a Source Data file.
Abbreviations: Dx: diagnosis; Norm: normal; Met: metastatic; mCRC: metastatic colorectal cancer; PAO: peritoneum-abdominal wall-omentum; LN: lymph node; Gyn: gynecologic.

Supplementary Figure 3

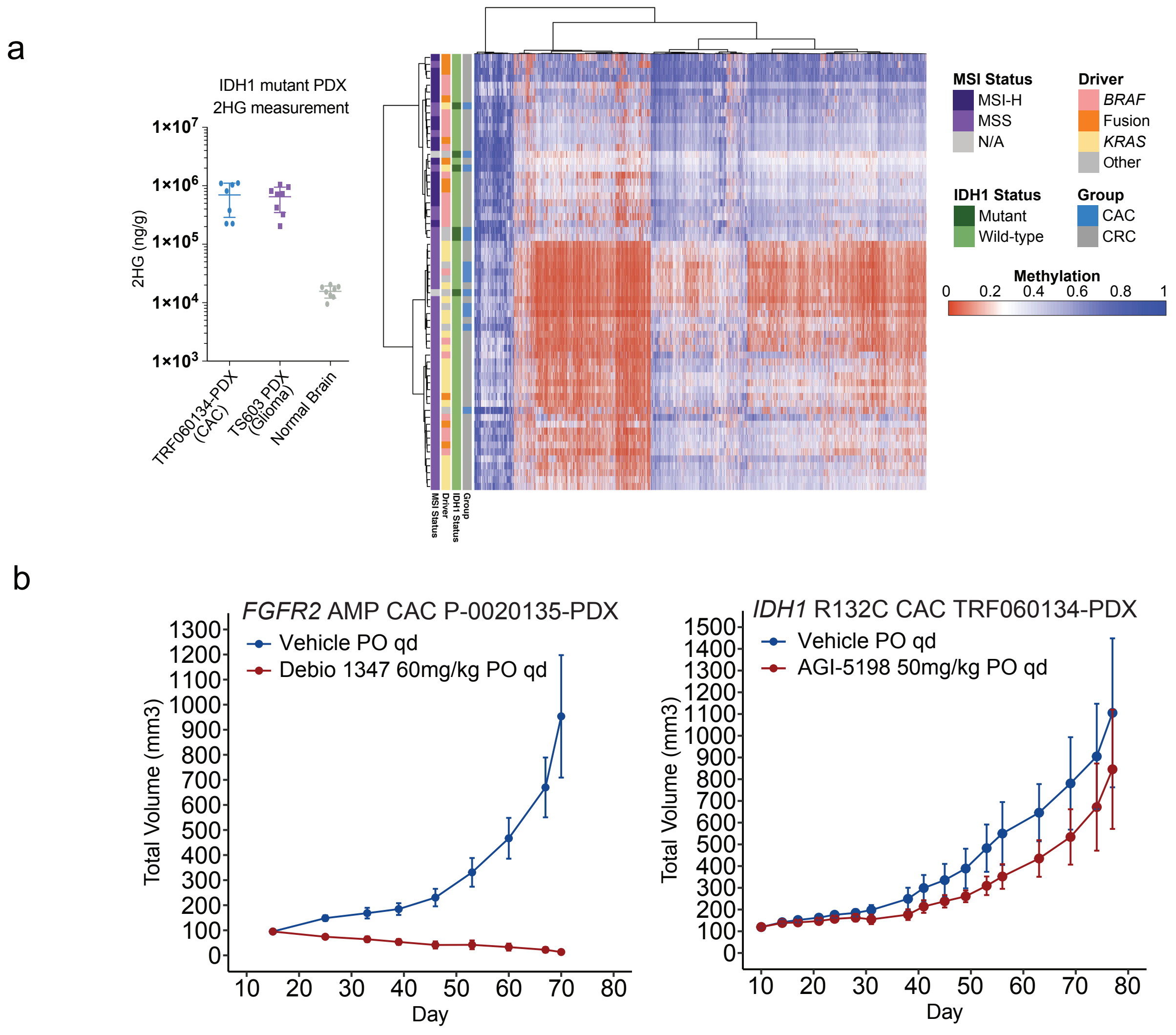

Supplementary Fig. 3. Functional effects of genomic alterations in CAC.
a, Levels of 2HG detected from *IDH1* mutant CAC patient-derived xenografts (PDXs) (n=7) , *IDH1* mutant glioma PDXs (n=8) , and normal brain tissue (n=8). The levels of 2HG were significantly higher in the CAC PDXs and glioma PDXs versus normal brain (P = 0.00031, P = 0.00016, respectively) based on a two-sided Mann–Whitney U-test. The center line of the boxplots indicates the median, the edges indicate the interquartile range, and the whiskers extend to the highest and lowest values not considered outliers. **b,** Growth curves of PDXs with *FGFR2* amplification (P = 0.01) or *IDH1* R132C mutation (P =0.21) (n = 5 per group) treated with matched targeted therapy. The center point indicates the mean while the and error bars show standard deviations in tumor volume. A two-sided Mann-Whitney U-test was used to assess statistical significance.
Source data are provided as a Source Data file.

Supplementary Figure 4

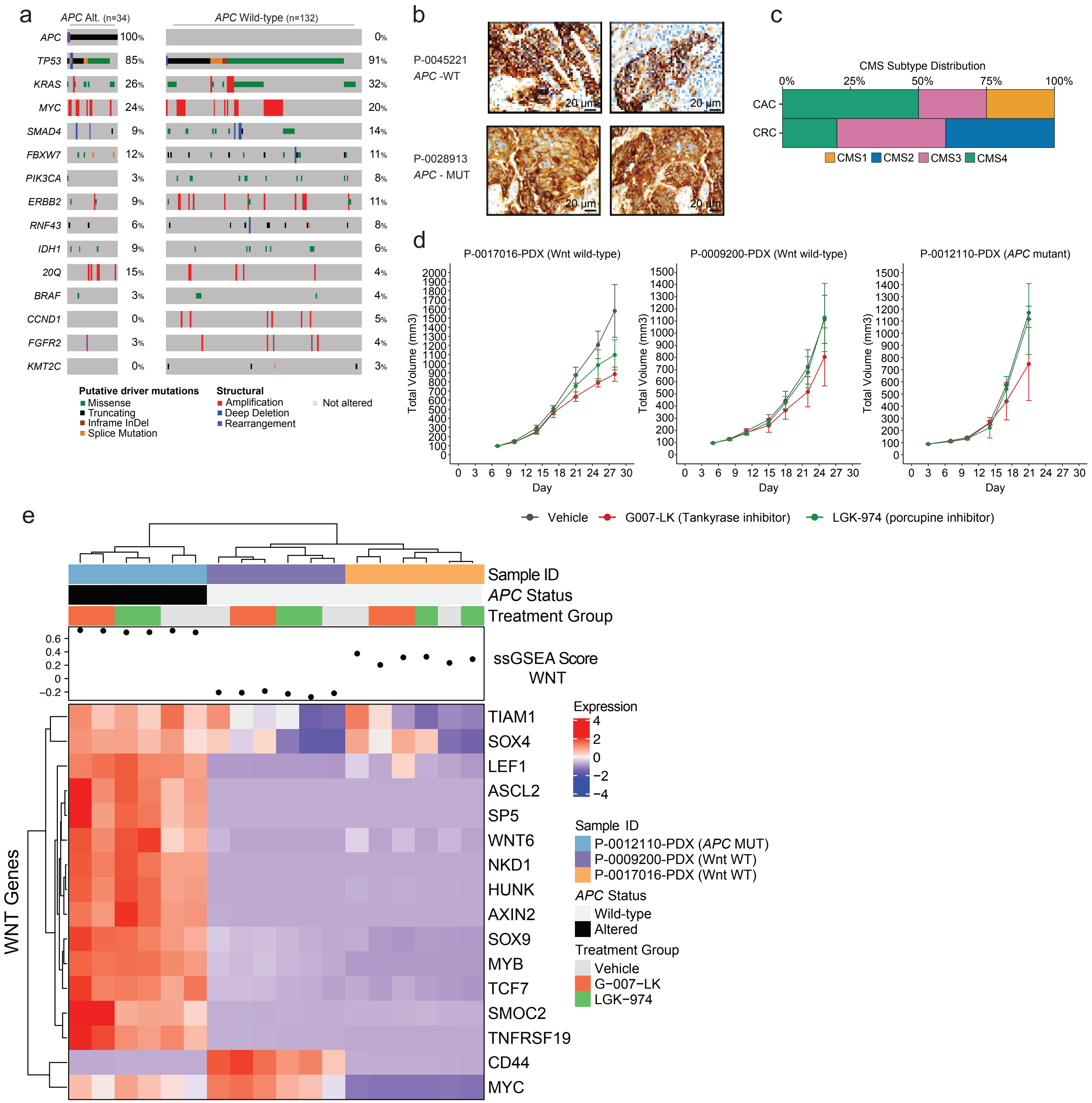

Supplementary Fig. 4. Wnt dependence of CAC.

a, Oncoprints of recurrently mutated genes in CAC by Wnt mutation status. **b,** Immunohistochemical assessment of beta-catenin expression in representative colitis-associated cancer tumor specimens. Experiment was conducted 14 times. **c,** Consensus molecular subtypes of sporadic colorectal (n = 6) and colitis-associated (n = 5) cancers subjected to RNA sequencing. **d,** Growth curves of CAC patient-derived xenografts grown in vehicle control, the Tankyrase inhibitor G007-LK, or the porcupine inhibitor LGK-974 (n = 5 mice per group). The center point indicates the mean while the error bars show standard deviations in tumor volume. A two-sided Mann-Whitney U-test was used to assess statistical significance. Statistical comparisons are as follows: P-0017016-PDX: vehicle versus G007-LK treatment, P = 0.01, vehicle versus LGK-974 treatment, P = 0.03; P-0009200 PDX: vehicle versus G007-LK treatment, P = 0.10, vehicle versus LGK-974, P =0.84; P-0012110 PDX: vehicle versus G007-LK treatment, P = 0.02, vehicle versus LGK-974 treatment, P = 0.84. **e,** Expression of Wnt pathway genes in patient-derived xenografts (n=18) collected at the end of the treatments. Source data are provided as a Source Data file.

Abbreviations: Alt: altered; WT: wild-type; MUT: mutated.

Supplementary Figure 5

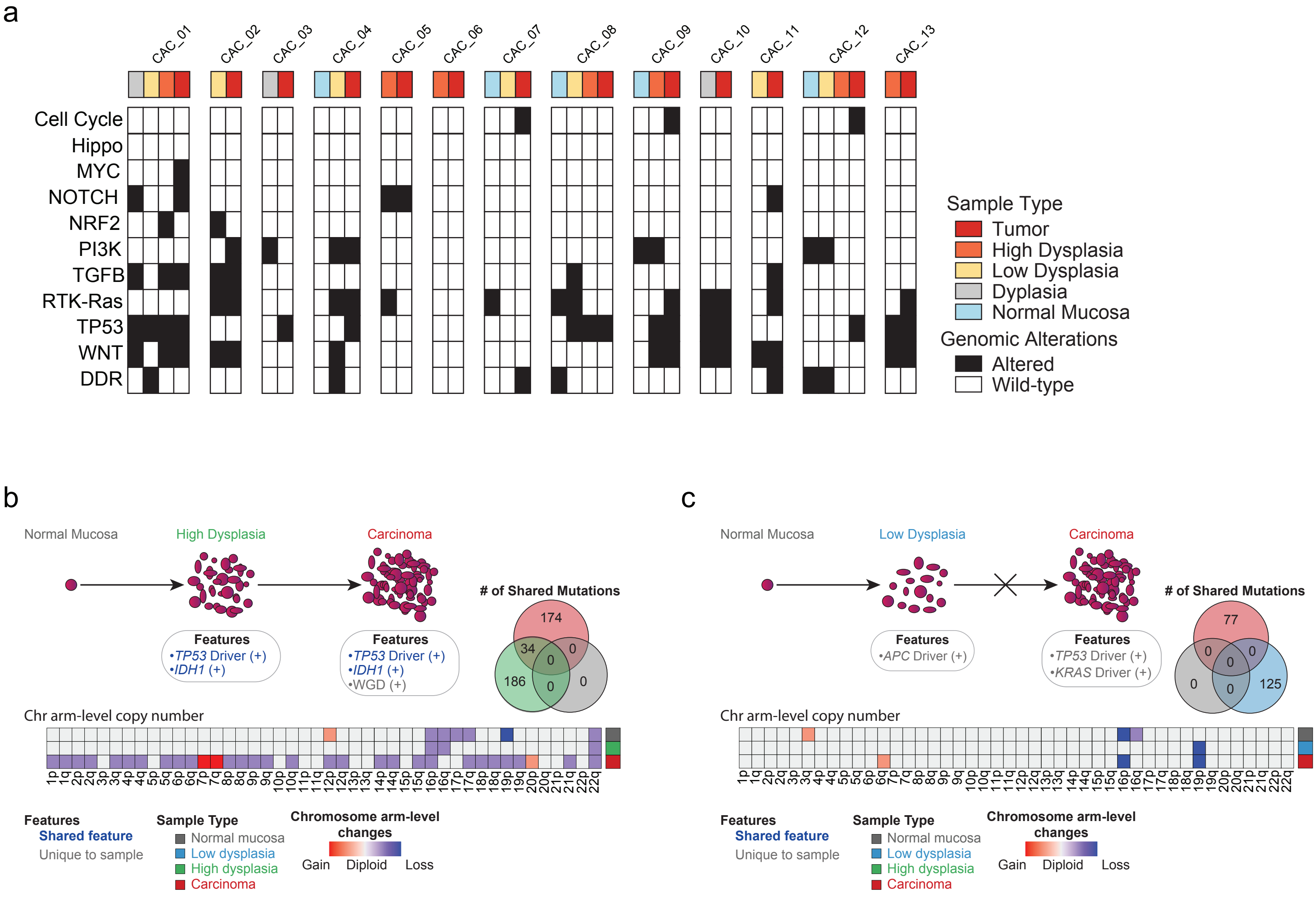

Supplementary Fig. 5. Genetic relatedness of dysplastic and cancerous lesions developing in patients with inflammatory bowel disease.
a, Pathway alterations identified by whole exome sequencing (WES) of samples from 16 patients with at least a single focus of colitis-associated dysplasia. In the 13 patients with multiple samples, all samples were collected at the same time. Patients with mucosa specimens indicated had alterations in mucosa identified at WES called against matched blood normal, and patients without indicated mucosa specimens had the mucosa manually reviewed for alterations in *TP53*, *APC*, and *KRAS* as matched blood (normal) was not available. **b,** Genomic alterations detected by WES and their relationship in samples of normal mucosa, high grade dysplasia, and carcinoma taken from a patient with ulcerative colitis. **c,** Genomic alterations detected by WES and their relationship in samples of normal mucosa, low grade dysplasia, and carcinoma taken from a patient with ulcerative colitis.
Source data are provided as a Source Data file.
Abbreviation: WT: wild-type.

References:

1. Richards, S. et al. Standards and guidelines for the interpretation of sequence variants: a joint consensus recommendation of the American College of Medical Genetics and Genomics and the Association for Molecular Pathology. Genet Med 17, 405-424 (2015).

2. Moghadasi, S. et al. Classification and Clinical Management of Variants of Uncertain Significance in High Penetrance Cancer Predisposition Genes. Hum Mutat 37, 331-336 (2016).

3. Yaeger, R. et al. Genomic Alterations Observed in Colitis-Associated Cancers Are Distinct From Those Found in Sporadic Colorectal Cancers and Vary by Type of Inflammatory Bowel Disease. Gastroenterology 151, 278-287.e6 (2016).
