# Supplementary material for: Integrated clinical and genomic analysis identifies driver events and molecular evolution of colitis-associated cancers
Source: Nat Commun. 2023 Jan 7;14:110. doi: 10.1038/s41467-022-35592-9 (PMC9825391; doi:10.1038/s41467-022-35592-9)
Supplement: Supplementary file 3 — Reporting Summary [file 41467_2022_35592_MOESM3_ESM.pdf]

Corresponding author(s): Rona Yaeger
Last updated by author(s): Nov 9, 2022

Reporting Summary

Nature Portfolio wishes to improve the reproducibility of the work that we publish. This form provides structure for consistency and transparency in reporting. For further information on Nature Portfolio policies, see our Editorial Policies and the Editorial Policy Checklist.

Statistics

For all statistical analyses, confirm that the following items are present in the figure legend, table legend, main text, or Methods section.

n/a	Confirmed	
☐	☒	The exact sample size (*n*) for each experimental group/condition, given as a discrete number and unit of measurement
☐	☒	A statement on whether measurements were taken from distinct samples or whether the same sample was measured repeatedly
☐	☒	The statistical test(s) used AND whether they are one- or two-sided<br>*Only common tests should be described solely by name; describe more complex techniques in the Methods section.*
☐	☒	A description of all covariates tested
☐	☒	A description of any assumptions or corrections, such as tests of normality and adjustment for multiple comparisons
☐	☒	A full description of the statistical parameters including central tendency (e.g. means) or other basic estimates (e.g. regression coefficient) AND variation (e.g. standard deviation) or associated estimates of uncertainty (e.g. confidence intervals)
☐	☒	For null hypothesis testing, the test statistic (e.g. *F*, *t*, *r*) with confidence intervals, effect sizes, degrees of freedom and *P* value noted<br>*Give P values as exact values whenever suitable.*
☒	☐	For Bayesian analysis, information on the choice of priors and Markov chain Monte Carlo settings
☐	☒	For hierarchical and complex designs, identification of the appropriate level for tests and full reporting of outcomes
☒	☐	Estimates of effect sizes (e.g. Cohen's *d*, Pearson's *r*), indicating how they were calculated

Our web collection on statistics for biologists contains articles on many of the points above.

Software and code

Policy information about availability of computer code

Data collection	No software was used for data collection.
Data analysis	Data from the MSK-IMPACT platform was processed and analyzed using custom scripts available through the MSK Github repository (https://gitbhub.com/mskcc). WES data were processed using the Roslin platform developed at the Center for Molecular Oncology at MSK (https://github.com/mskcc/roslin-variant/wiki). Analysis of copy number alterations were performed for both MSK-IMPACT and WES sequenced samples with available matched normal using version 0.5.14 of the FACETS (Fraction and Allele-Specific Copy Number Estimates form Tumor Sequencing) tool (https://github.com/mskcc/facets). CNApp was used to analyze tumor sample segmentation data and generate both focal and broad CNA scores (https://tools.idibaps.org/CNApp/). OncoKB software (oncokb-annotator v1.0.7) was used to annotate mutations (https://github.com/oncokb). Analysis of differential gene expression was conducted using DSeq2 v.1.30.165. Single sample gene set enrichment analysis (ssGSEA) was performed using the R package GSVA 67. All statistical analyses were performed using R v3.5.2 (www.R-project.org) and Bioconductor v3.4.

For manuscripts utilizing custom algorithms or software that are central to the research but not yet described in published literature, software must be made available to editors and reviewers. We strongly encourage code deposition in a community repository (e.g. GitHub). See the Nature Portfolio guidelines for submitting code & software for further information.

Data

Policy information about availability of data

All manuscripts must include a data availability statement. This statement should provide the following information, where applicable:

- Accession codes, unique identifiers, or web links for publicly available datasets
- A description of any restrictions on data availability
- For clinical datasets or third party data, please ensure that the statement adheres to our policy

All clinical and genomic sequencing data described in this manuscript have been deposited in the cBioPortal for Cancer Genomics and are publicly available for online browsing and bulk download through the following link: http://www.cbioportal.org/study/summary?id=bowel_colitis_msk_2022. The raw sequencing data are protected; de-identified data are available under restricted access to protect patient privacy in accordance with federal and state law. These data can be requested for research use from the corresponding author, subject to institutional approvals. Additionally, all clinical data and variant call data are included in the Source Data File. The raw RNA sequencing data generated in this study have been deposited in GEO (accession number GSE### available at XX). All other data generated in this study are available within the article and its supplementary data files. Source data are provided with this paper.

Human research participants

Policy information about studies involving human research participants and Sex and Gender in Research.

Reporting on sex and gender	Participant sex is included in the Source Data file.
Population characteristics	Population characteristics are summarized in Table 1.
Recruitment	Patients with colitis-associated cancers were identified from Memorial Sloan Kettering Cancer Center (MSK) (n=130), Weill Cornell Medical Center-New York Presbyterian Hospital (WCMC) (n=29), or Sheba Medical Center in Israel (n=7). Samples were initially identified retrospectively through a query and validation of the pathology databases of these institutions for cases where colitis was a clinical factor noted in the pathology report. Since 2015, cases have been identified prospectively at MSK through a biweekly computerized query for any patients with "colitis" as a diagnostic term who is scheduled to be seen in the surgical, gastroenterology, or medical oncology clinics. There may be a bias for patients with more advanced disease presenting to MSK. We have summarized the stage distribution of cancers studies and assessed for associations between genomic changes and tumor stage. We don't believe this potential bias impacted the reported results as we identified no significant associations in genomic alterations in colitis-associated cancers by tumor stage.
Ethics oversight	The study was approved by the Memorial Sloan Kettering Cancer Center Institutional Review and Privacy Board.

Note that full information on the approval of the study protocol must also be provided in the manuscript.

Field-specific reporting

Please select the one below that is the best fit for your research. If you are not sure, read the appropriate sections before making your selection.

☒ Life sciences ☐ Behavioural & social sciences ☐ Ecological, evolutionary & environmental sciences

For a reference copy of the document with all sections, see nature.com/documents/nr-reporting-summary-flat.pdf

Life sciences study design

All studies must disclose on these points even when the disclosure is negative.

Sample size	We have included all dysplasia and colitis-associated cancers available for genomic analysis. We have assembled the largest series of colitis-associated cancers analyzed to date.
Data exclusions	Patients with Lynch syndrome or microsatellite instable tumors were excluded to focus on the role of inflammation in cancer development. These exclusion criteria were pre-established for the data analysis.
Replication	Genomic findings in patients' tumor samples were analyzed in patient-derived models to understand their functional effect. Multiple samples were analyzed, and multiple patient-derived organoids and xenografts were studied. Patient-derived xenograft experiments were performed with at least 5 mice per group with growth curves showing mean and standard deviation measurements. Organoid growth experiments were performed with at least four biological replicates and mean and standard deviations are presented. Photographs of organoid growth are representative images from experiments conducted with three replicates. The patient-derived xenograft and organoid models used in this manuscript were established before the growth experiments commenced and all replication attempts for the reported experiments were successful.
Randomization	This is an observational study of genomic and germline changes and gene expression in neoplastic lesions collected from patients with colitis-associated dysplasia or cancer. Patients were not randomized in this study. In the in vivo experiments, mice were randomized to achieve similar average tumor size in every treatment arm receiving drug treatments or vehicle as control.

Blinding	No blinding was performed as this is an observational study of genomic and germline changes and gene expression in neoplastic lesions collected from patients with colitis-associated dysplasia or cancer.

Reporting for specific materials, systems and methods

We require information from authors about some types of materials, experimental systems and methods used in many studies. Here, indicate whether each material, system or method listed is relevant to your study. If you are not sure if a list item applies to your research, read the appropriate section before selecting a response.

Materials & experimental systems

n/a	Involved in the study	
☐	☒	Antibodies
☒	☐	Eukaryotic cell lines
☒	☐	Palaeontology and archaeology
☐	☒	Animals and other organisms
☐	☒	Clinical data
☒	☐	Dual use research of concern

Methods

n/a	Involved in the study	
☒	☐	ChIP-seq
☒	☐	Flow cytometry
☒	☐	MRI-based neuroimaging

Antibodies

Antibodies used	IHC for β-catenin was performed on a BenchMark XT automated immunostainer (Ventana Medical Systems Inc., Tucson, AZ). Sections were incubated with anti-β-catenin antibody (Cell Marque, catalog #760-4242) at a concentration of 1.73ug/mL. Antigen retrieval was performed with Cell Conditioning Solution (CC1, Ventana Medical Systems Inc.) for 24 hours, and primary antibody incubation was for 24 hours. Antigen detection was performed using the Optiview DAB Detection kit (Ventana Medical Systems Inc.).
Validation	All the antibodies were validated by manufacturers. The primary antibody used is a mouse monoclonal antibody optimized for immunohistochemical staining of formalin-fixed, paraffin-embedded tissue sections.

Animals and other research organisms

Policy information about studies involving animals; ARRIVE guidelines recommended for reporting animal research, and Sex and Gender in Research

Laboratory animals	4-6-week-old NSG female mice used for PDX experiments. 8-10-week old male wild-type C57BL/6J mice were used to generate AOM/DSS mouse model of inflammatory colon cancer.<br>Animals were housed in individually ventilated caging systems (model nos. 19-140-10-14-1-4-7TMAL and 9-140-10-14-1-4-7TMAL, Thoren Caging Systems, Hazleton, PA), whose effluent was exhausted directly into the building's HVAC system, on autoclaved aspen chip bedding (PJ Murphy Forest Products, Montville, NJ) and were provided a γ-irradiated commercial diet (PicoLab Rodent Diet 20, 5053 LabDiet, PMI Nutrition International, St Louis, MO), and acidified water (pH 2.5 to 2.8) ad libitum. Mice were housed at a population density that ranged from 1 to 5 mice per cage in an environment providing a temperature of 21.1 to 22.2 °C (70 to 72 °F), 30% to 70% humidity, 10 to 15 fresh air exchanges hourly, and a 12:12-h light:dark cycle (lights on, 0600 to 1800).
Wild animals	The study did not involve wild animals.
Reporting on sex	Female mice were included in the in vivo drug experiments; male mice were used to generate the AOM/DSS mouse model of inflammatory colon cancer. Results are not anticipated to vary by sex, and differences in alterations in Wnt pathway genes in clinical specimens did not vary by sex.
Field-collected samples	The study did not involve samples collected on the field.
Ethics oversight	Studies were performed in compliance with institutional guidelines under an IACUC approved protocol (MSKCC and Dana Farber Cancer Institute).

Note that full information on the approval of the study protocol must also be provided in the manuscript.

Clinical data

Policy information about clinical studies

All manuscripts should comply with the ICMJE guidelines for publication of clinical research and a completed CONSORT checklist must be included with all submissions.

Clinical trial registration	N/A
Study protocol	N/A
Data collection	N/A

Outcomes	N/A
